# Chicago Veterinary College

**Published:** 1893-12

**Authors:** 


CHICAGO VETERINARY COLLEGE.

*March 24th.—Degree M. D. C. (Doctor of Comparative Medicine.)*

| | |
|---|---|
| Adamson, J. H. | Norfolk, Va. |
| Anderman, F. W. | Chicago, Ill. |
| Armstrong, G. E. | Sanborn. Ia. |
| Babb, A. | Springfield, Ill. |
| Barrett, Dan | Cascade, Ia. |
| Baxter, C. E. | Griswold, Ia. |
| Bennett, G. M. | Chicago, Ill. |
| Binger, S. | Monroe, Wis. |
| Bovett, J. A. | Chicago, Ill. |
| Bruette, W. A. | Chicago, Ill. |
| Casper, A. M. | Milwaukee, Wis. |
| Casserly, W. H. | St. Paul, Minn. |
| Clark, W. G. | Johnstown, Wis. |
| Cobb, G H., Jr. | Corning, N. Y. |
| Cole, W. H. | Kewanee, Ill. |
| Crane, C. M. | Waukesha, Wis. |
| Davis, F. H. | Ensworth, Wis. |
| Deenis, C. G. | Ottawa, Ill. |
| Downs, N. H. | Cleveland, O. |
| Draper, O. G. | Macon, Mo. |
| Durack, J. D. | Mineral, Ill. |
| Eaton, R. D. | Minneapolis, Minn. |
| Eckley. M. C. | Woodhull, Ill. |
| Eddy, J. H. | Stockton, Cal. |
| Everton, C. G. | Monroe Center, Ill. |
| Faulkner, G. F. | Salinas, Cal. |
| Fay, G. H. | Oakfield, Wis. |
| Goodale, O. | Toulon, Ill. |
| Gould, J. N. | Fairmount, Minn. |
| Gould, J. W. | Fairmount, Minn. |
| Gwinn, W. T. | Newman, Ill. |
| Gysel, R. | So. Chicago, Ill. |
| Hagadone, G. L. | Chicago, Ill. |
| Herzer, E. | Milwaukee, Wis. |
| Higgins, R. A. | Milwaukee, Wis. |
| Hill, Geo. C. | Milwaukee, Wis. |
| Hill, M. V. | Minnesota Lake, Minn. |
| Ilstrup, F. A. | Buffalo, Minn. |
| Kaylor, J. M. | Barry, Ill. |
| Kermath, D. | Chicago, Ill. |

| | |
|---|---|
| Ketchum, F. D. - - - - | Marseilles, Ill. |
| Koehne, Chas. - - - | Paynesville, Wis. |
| Lamont, F. G. - - - - | Esmond, Ill. |
| Leith, F. J. - - - - - | Chicago, Ill. |
| Longnecker, A. O. - - - - | Atlanta, Ill. |
| McAllister, J. H. - - - - | Lee, Mass. |
| McDonald, J. T. - - - - | Taylorville, Ill. |
| McGraw, E. F. - - - - | Ft. Scott, Kas. |
| McKenna, C. S. - - - - | Mt. Morris, N. Y. |
| McNair, F. S. - - - - | Elburn, Ill. |
| McNay, G. P. - - - - | Humeston, Ia. |
| Merrick, C. H. - - - | Okawville, Ill. |
| Metzger, A. E. - - - - | Clyde, O. |
| Morgenroth, E. L. - - - | Boltonville, Wis. |
| Mullett, J. H. F. - - - | Cassopolis, Mich. |
| Newbury, M. C. - - - | Hanover, Mich. |
| Newton, E. H. - - - - | Poynette, Wis. |
| Oberst, J. J. - - - - | Belgium, Wis. |
| Presler, H. A. - - - | Montpelier, O. |
| Rich, R. G. - - - - - | Fayette, Ia. |
| Richmond, F. O. - - - - | Sabetha, Kas. |
| Rimmer, F. - - - - - | Chicago, Ill. |
| Rimmer, T. - - - - | Chicago, Ill. |
| Rork, A. M. - - - - | Merrill, Wis. |
| Rushworth, W. A. - - - | Fort Collins, Colo. |
| Sawyer, F. N. - - - - | Chicago, Ill. |
| Schoedde, B. - - - | Beckwith, Wyo. |
| Scott, J. A. - - - - | Minneapolis, Minn. |
| Sheppard, J. N. - - - | Park River, N. D. |
| Stanley, O. W. - - - - | Sioux Falls, S. D. |
| Stevens, C. W. - - - - | Knoxville, Ia. |
| Sutzin, J. - - - - | Minneapolis, Minn. |
| Thornborrow, J. A. - - - | Jacksonville, Ill. |
| Troxell, R. E. - - - - | Chicago, Ill. |
| Ulm, F. J. - - - - | Chicago, Ill. |
| Van Aken, R. D. - - - | Columbus, Wis. |
| Waterman, G. A. - - - - | Storrs, Conn. |
| Wheeler, E. G. - - - - | Pella, Ia. |
| White, S. J. - - - - | E. St. Louis, Ill. |
| Wiesen, W. J. - - - - | Chicago, Ill. |
| Wright, F. O. - - - - | Smithport, Pa. |
| Ziegenhorn, A. F. - - - - | Claytonville, Ill. |

